# Immune Checkpoint Inhibitor Induced Diabetes Mellitus Treated with Insulin and Metformin: Evolution of Diabetes Management in the Era of Immunotherapy

**DOI:** 10.1155/2019/8781347

**Published:** 2019-11-04

**Authors:** Taha Alrifai, Faisal Shaukat Ali, Sameer Saleem, Diana Carolina Miranda Ruiz, Dana Rifai, Sundas Younas, Faisal Qureshi

**Affiliations:** ^1^Department of Internal Medicine, AMITA Health Saint Joseph Hospital, Chicago, IL 60657, USA; ^2^Department of Internal Medicine, Saint Catherine Hospital, East Chicago, IN 46312, USA; ^3^Khyber Medical College, Peshawar, Pakistan; ^4^Department of Endocrinology, AMITA Health Saint Joseph Hospital, Chicago, IL 60657, USA

## Abstract

Immune checkpoint inhibitors (ICPIs) are a breakthrough therapy in oncology and have been approved by the Food and Drug Administration for the treatment of several malignancies. ICPIs have been reported to cause immune-mediated damage of islet cells leading to ICPI-induced type 1 diabetes mellitus (T1DM). These reports described patients presenting with severe diabetic ketoacidosis (DKA). We present a case of a 69-year-old Caucasian male with type 2 diabetes suffering from non-small cell lung cancer and undergoing treatment with pembrolizumab, an anti-programmed cell death protein-1 antibody, who presented to our emergency department with complaints of nausea, vomiting, polyuria, and polydipsia. He was found to have high anion gap metabolic acidosis with ketosis and elevated blood glucose levels consistent with DKA. Lab workup was consistent with T1DM. Despite being on a tailored insulin regimen, his blood glucose remained elevated, necessitating the addition of metformin to his regimen which effectively controlled his blood glucose.

## 1. Background

Since the approval of immune checkpoint inhibitors (ICPIs), several case reports have emerged describing adverse outcomes pertaining to the activation of patients' immune systems. These immune-related adverse events (irAEs) can theoretically affect any organ system [1]. Several case reports have been published describing new onset diabetes mellitus in cancer patients receiving programmed cell death protein-1 (PD-1) inhibitors which is thought to be a consequence of immune-mediated beta islet cell dysfunction, resulting in a type 1 diabetes mellitus (T1DM) [2–6]. However, data regarding management of new onset T1DM, particularly among patients with a history of T2DM, is scarce. We report a case of a 69-year-old cancer patient with a history of T2DM who developed T1DM after undergoing treatment with pembrolizumab, a PD-1 inhibitor, which was effectively managed with insulin and metformin.

## 2. Case Presentation

A 69-year-old male with a medical history of T2DM and stage IV NSCLC presented to our ED with complaints of nausea, vomiting, polyuria, polydipsia, and weakness of 4 days duration. His T2DM was diagnosed 4 years prior to his presentation and had been managed with oral antidiabetic drugs (OADs); his glycohemoglobin levels ranged between 5.9 and 7.3%. He had been undergoing chemotherapy with carboplatin, pemetrexed, and pembrolizumab and had completed his 4^th^ cycle 20 days prior to this presentation.

On physical examination, he had dry oral mucosa and was tachypneic with a respiratory rate of 30 respirations per minute. Laboratory workup revealed a pH of 6.95, a serum bicarbonate level of 3 mmol/L with an anion gap of 39 mmol/L. Serum osmolality was elevated at 342 mOsm/kg. Blood glucose was 907 mg/dL, and serum ketones were 5.3 mmol/L. His serum potassium level was 7.5 mmol/L, and his serum creatinine level was elevated to 2.72 mg/dL from a normal baseline. Glycohemoglobin was 9.2%. The patient was admitted to the intensive care unit (ICU), where he was started on an intravenous (IV) insulin infusion and IV fluids with serial monitoring of his blood glucose, pH, and potassium levels. His DKA resolved and he was started on long-acting and short-acting insulin.

Workup for potential precipitating events leading to DKA was done. After exclusion of infectious and common metabolic etiologies, it was thought that the patient's treatment with pembrolizumab may have led to new-onset ICPI-induced T1DM presenting with DKA. Subsequently, C-peptide levels were checked and were undetectable (<0.1 mg/mL). Anti-glutamic acid decarboxylase antibody (Anti-GAD) titers were elevated at 61 U/mL, both of which were consistent with a diagnosis of T1DM. His pembrolizumab was held for one cycle and resumed thereafter.

Following his discharge from the hospital, the patient was maintained on a regimen of long- and short-acting insulin with insulin degludec and insulin aspart. However, it became challenging to achieve adequate glycemic control despite the patient's compliance with insulin therapy. His glycohemoglobin was persistently high and was 8.7% after being on insulin for 15 months. His insulin regimen was subsequently supplemented with metformin, which led to a decrease in his glycohemoglobin from 8.7% to 7.7% one month after the change in regimen. He remains on maintenance therapy with pembrolizumab for his NSCLC and has not had further ICPI-associated complications on his last follow-up.

## 3. Discussion

ICPIs evolved the paradigm of cancer therapy, proving efficacious for a myriad of cancers including metastatic melanoma, NSCLC, and renal cell carcinoma, with a plethora of clinical trials underway to assess the safety and efficacy of this drug class in various other cancers [7]. Owing to the immune-mediated mechanism of action of ICPIs, they have led to a wide spectrum of irAEs. ICPI-induced islitis and subsequent development of ICPI-induced T1DM or evolution of T2DM into ICPI-induced T1DM is a rare entity and may be difficult to identify upon presentation. The first case of ICPI-induced T1DM was reported in 2015 with the use of pembrolizumab [8]. Subsequently, multiple case reports of ICPI-induced T1DM associated with the use of PD-1 inhibitors [2–6], as well as cytotoxic T-lymphocyte-associated protein-4 (CTLA-4) inhibitors [9], emerged, shedding light on this uncharacterized entity. To date, the largest case series are comprised of less than 30 patients, highlighting the scarcity of data concerning ICPI-induced T1DM [10].

Immune-mediated islet injury has been thought to be a consequence of T-cell modulation, evident by the presence of GAD-65 antibodies, though these have not been reported in all cases of ICPI-induced T1DM to date, highlighting the inadequacy of the T-cell immunomodulation theory [11]. However, an upregulation of CD-8 T-cell-mediated response to T1DM antigens, as has been reported in a fraction of cases, weighs in favor of a T-cell-mediated injury. Nonetheless, the current hypotheses identify pieces of the puzzle which, when put together, may provide an all-inclusive understanding of the mechanisms behind this rare entity.

Serial blood glucose monitoring of patients being treated with ICPIs on regular intervals is crucial for recognition of ICPI-induced T1DM. In patients with a history of T2DM, ICPI-induced T1DM may be masked and overlooked as a manifestation of uncontrolled T2DM, highlighting the importance of a low threshold for suspicion and initiation of further workup. Wright et al. suggested obtaining GAD antibody levels in patients with T2DM prior to initiating therapy with ICPI to identify those who might be at risk for developing immune-mediated DM [12]. Patients with ICPI-induced T1DM which has gone unrecognized may develop DKA which is potentially fatal if not recognized and treated in a timely manner. The National Comprehensive Cancer Network (NCCN) recommends holding ICPI therapy among patients who present with a blood glucose of >250 m/dL or DKA, as was done in our case [13].

The management of ICPI-induced T1DM is not well characterized and is currently stemming from our understanding of T2DM, T1DM, and DKA. ICPI-induced insulin deficiency may warrant therapy with a tailored insulin regimen, as is done for patients with T1DM. However, among patients with a history of T2DM, this may not address the insulin resistance, highlighting the need for studies addressing the management of ICPI-induced T1DM. Among patients with a history of T2DM, the treatment of ICPI-induced T1DM may constitute a combination of OACs to tackle insulin resistance, along with a customized insulin regimen to address the acquired insulin deficiency. The OACs which our patient was taking prior to presentation were held temporarily and he was started on insulin degludec and insulin aspart. However, due to his history of T2DM and difficulty in achieving adequate glycemic control despite being on a tailored insulin regimen for 15 months, metformin was reintroduced to his regimen which led to a noticeable improvement in glycemic control. Hence, ICPI-induced T1DM among patients with a history of T2DM represents a new entity requiring an evolved treatment approach where a baseline insulin regimen may be supplemented with OACs such as metformin to counteract insulin resistance and achieve better glycemic control.

Several irAEs have been reported to spontaneously resolve with the discontinuation of immunotherapy [14]. This might not prove true for the majority of ICPI-induced endocrinopathies [15]. Further large-scale studies concerning the reversibility of ICPI-induced endocrinopathies are urgently needed.

In conclusion, ICPI-induced T1DM is a rare irAE. Clinicians involved in the care of cancer patients on ICPI therapy should be mindful and maintain a high clinical index of suspicion for this rare entity. In patients with preexisting T2DM, ICPI-induced T1DM can be treated with an insulin regimen supplemented with OACs to counter the insulin resistance and achieve better glycemic control. Further large-scale studies are needed to characterize the management of ICPI-induced T1DM in patients with preexisting T2DM further.
